# The potential role of exosomal circRNAs in the tumor microenvironment: insights into cancer diagnosis and therapy

**DOI:** 10.7150/thno.64096

**Published:** 2022-01-01

**Authors:** Juan Li, Guo Zhang, Chen-Guang Liu, Xiaoqiang Xiang, Minh T.N. Le, Gautam Sethi, Lingzhi Wang, Boon-Cher Goh, Zhaowu Ma

**Affiliations:** 1 Key Laboratory of Environmental Health, Ministry of Education, Department of Toxicology, School of Public Health, Tongji Medical College, Huazhong University of Science and Technology, Wuhan, Hubei 430030, China.; 2 School of Basic Medicine, Health Science Center, Yangtze University, 1 Nanhuan Road, Jingzhou, Hubei 434023, China.; 3 Department of Clinical Pharmacy and Pharmacy Administration, School of Pharmacy, Fudan University, Shanghai 201203, China.; 4 Department of Pharmacology, Yong Loo Lin School of Medicine, National University of Singapore, 117600 Singapore.; 5 Cancer Science Institute of Singapore, National University of Singapore, 117599 Singapore.; 6 Department of Haematology-Oncology, National University Cancer Institute, Singapore 119228, Singapore.

**Keywords:** Exosome, circular RNAs, tumor microenvironment, diagnosis, cancer therapy.

## Abstract

Exosomes are multifunctional regulators of intercellular communication by carrying various messages under both physiological and pathological status of cancer patients. Accumulating studies have identified the presence of circular RNAs (circRNAs) in exosomes with crucial regulatory roles in diverse pathophysiological processes. Exosomal circRNAs derived from donor cells can modulate crosstalk with recipient cells locally or remotely to enhance cancer development and propagation, and play crucial roles in the tumor microenvironment (TME), leading to significant enhancement of tumor immunity, metabolism, angiogenesis, drug resistance, epithelial mesenchymal transition (EMT), invasion and metastasis. In this review, we describe the advances of exosomal circRNAs and their roles in modulating cancer hallmarks, especially those in the TME. Moreover, clinical application potential of exosomal circRNAs in cancer diagnosis and therapy are highlighted, bridging the gap between basic knowledge and clinical practice.

## Background

Recently, exosome biology and its clinical significance in oncology have attracted increasing attention. Exosomes are small extracellular vesicles (sEVs) that originate from intraluminal vesicles (ILVs) in the endosomal system, and are secreted from various cells upon fusion of multivesicular bodies with the plasma membrane 1. As such, exosomes can be detected in various bodily fluids, including blood, urine, saliva, and breast milk, thereby carrying a wide repertoire of cellular components from their parental cells, including proteins, lipids, DNAs, mRNAs, and noncoding RNAs (ncRNAs) 2. These exosomal cargoes can be delivered from their parental cells to recipient cells 3, 4. Exosome-mediated interplays between cancer cells and surrounding cells remodel the tumor microenvironment (TME), thereby creating favorable conditions for cancer progression and metastasis 5, 6.

Circular RNAs (circRNAs), which are abundant and become more stable in exosomes, can be transferred to neighboring or distant cells where they exert their functions 7. Exosomal circRNAs originating from cancer cells can be delivered to the recipient cells where they play vital roles in cancer cell initiation, progression, and proliferation, invasion and metastasis, as well as drug resistance 8. The relative stability, high abundance, and conserved nature across species of exosomal circRNAs make them potential biomarkers for various diseases, including cancers. Previous reports have illustrated the functions of various circRNAs in the TME, a complicated ecosystem incorporating the coevolution of both tumor cells and the surrounding stroma 9, 10. Dynamic alterations in the oncogenic components, including circRNAs and other TME features such as acidosis and hypoxia, play a crucial role in tumorigenesis and metastasis 11. In recent years, the focus on cancer therapy has shifted away from targeting malignant cancer cells to mediating the TME to interfere their complicated crosstalk. Hence, the TME has been considered a promising therapeutic target for cancer treatment 12.

Several reviews have discussed the roles of exosomal circRNAs in human diseases, including cancer 13-15, which focused on the functions and potential applications in different cancer types. Nevertheless, the role of exosome-derived circRNAs in regulating the TME has not been systematically summarized. Currently, targeting vital components of the TME is very attractive research areas for cancer therapy 10. Understanding the exosomal circRNA-mediated TME network may boost the development of therapies for diverse cancers. Herein, we conducted a comprehensive literature search to focus on latest research progress in the role of exosomal circRNAs in regulating the TME through affecting tumor immunity, tumor progression and metastasis, angiogenesis, drug resistance, and tumor metabolism. Furthermore, potential clinical applications of exosomal circRNAs as cancer biomarkers or therapeutic targets in cancer therapy have been discussed.

## Emerging roles of exosomes and circRNAs in cancer biology

### Characteristics of exosomes

**Figure 1:**
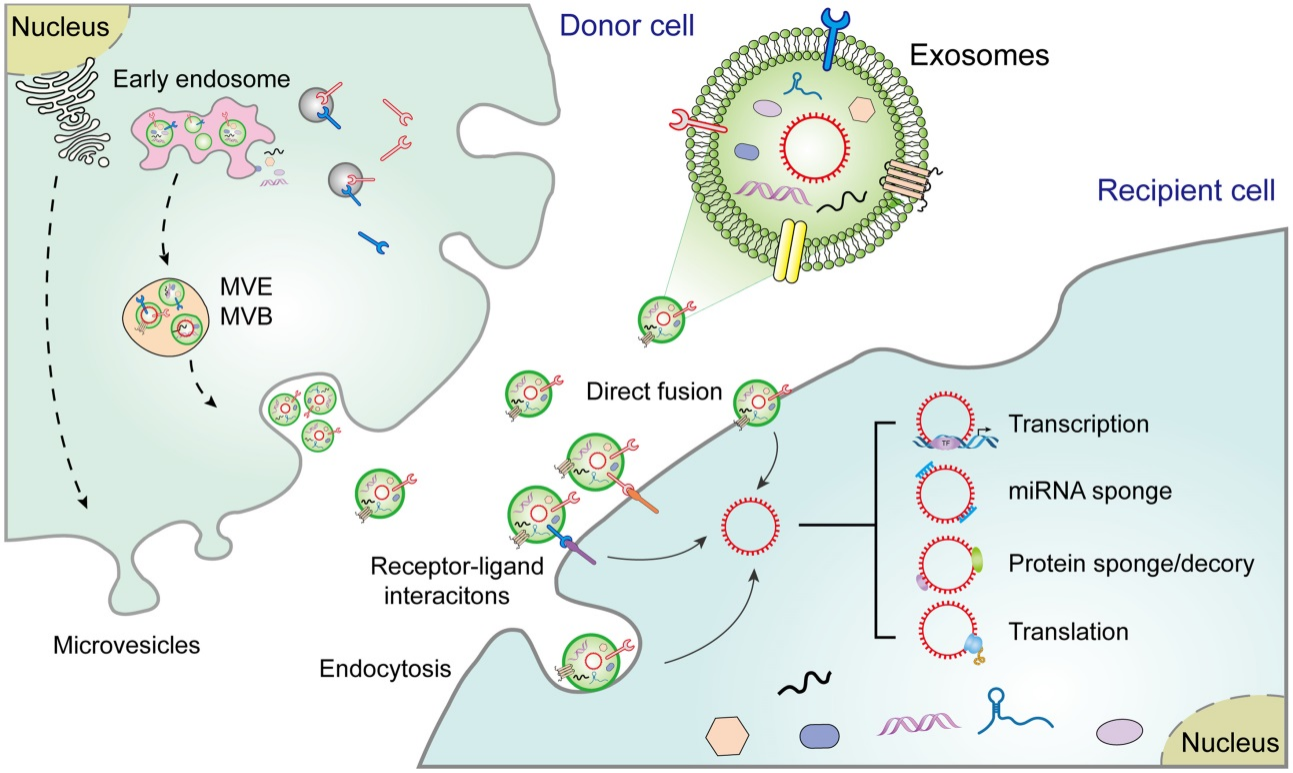
**Extracellular vesicle biogenesis and secretion in donor cells and their functions in recipient cells**. Exosomes participate in cell-to-cell communication via transferring diverse molecules, including exosomal circRNAs, new players in cancer diagnosis and therapy. Exosomes contribute to the interplay between donor cells and recipient cells through a variety of pathways, including cell-cell interactions, receptor-ligand interactions, and endocytosis. MVE: multivesicular endosome, MVB: Multivesicular body.

The biogenesis of EVs, including exosomes, microvesicles, and apoptotic bodies, is relatively well documented 16. Exosomes, the sEVs in diameter (40-150 nm), are secreted from diverse cell types 17. Their biogenesis begins with the formation of endosomes via endocytosis of the plasma membrane, followed by maturation of early endosomes into multivesicular endosomes/bodies (MVEs and MVBs). Exosomes are intraluminal vesicles (ILVs) produced within the endolysosomal system and shaped by the inward budding of the limiting membrane of MVEs/MVBs. Exosome formation involves a range of ordered molecular machineries, the most important of which is the endosomal sorting complexes required for transport (ESCRT), which is a key driver of membrane formation and scission, thus playing a crucial role in the formation of ILVs and MVEs (**Figure 1**) 3. Although exosomes were originally considered to be "garbage bags", functioning to extrude obsolete components from the cells, evidence suggests that they are in fact crucial regulators of the physiological or pathological processes by transferring biomolecules to recipient cells 3. For instance, once released, exosomes may act directly on neighboring, or distant recipient cells via various mechanisms, including ligand/receptor interaction, direct membrane fusion, and endocytosis, thus orchestrating multiple cellular processes in recipient cells 4. Alternatively, exosomes may merge with the membrane of a recipient cell and transfer their constituents into its cytosol 16. However, although exosomes share common characteristics with progenitor cells, they also contain distinct content that makes them unique 18.

### Exosomal circRNAs: emerging players in the TME

CircRNAs, a type of endogenous ncRNAs, are produced by a particular form of alternative splicing called back-splicing 19. CircRNAs were first discovered in viroids in 1976, and later considered as by-products of splicing errors 20. However, recent studies have highlighted the underlying importance of circRNAs while also characterizing their biogenesis and functions. CircRNAs have high abundance, conservation stability, and prevalence. Interestingly, unlike other RNAs, the absence of 5′ caps and 3′ tails allows for circRNAs to resist RNases and be more stable than linear RNAs. Hence, circRNAs may aggregate in cells to cause pathologies such as cancer progression 21. Therefore, circRNAs may become valuable diagnostic biomarkers or potential therapeutic targets for cancer treatment. Exosomes have been found to contain abundant diverse RNA species (e.g., circRNAs), and exosomal circRNAs contribute to tumorigenesis and progression via diverse mechanisms, including transcription, miRNA sponge, protein sponge/decoy, and translation (**Figure 1**). Therefore, studies involved in exosomal circRNAs have become a hotspot in intercellular communication in the TME, which is a complicated scaffold of stromal cells, extracellular matrix (ECM) components, and exosomes 22. The stromal cells include endothelial cells, pericytes, cancer-associated fibroblasts (CAFs), and immune cells, such as various types of lymphocytes, regulatory T cells (Treg), natural killer (NK) cells, tumor-associated macrophages (TAMs), and matrix metalloproteinases (MMPS), myeloid-derived suppressor cells chemokines, integrins, and other secreted molecules 22, 23. The tumor-derived exosomes (TDEs) contain various signaling molecules that mediate intercellular crosstalk and reprogramming of the TME 24. Thus, a thorough understanding of the phenotypic and functional characteristics in the TME may provide insights into novel treatment strategies for a diverse range of cancers.

The TME orchestrates cell-cell interactions through a various signaling networks, such as paracrine and juxtracrine interactions 23. Exosomes are excreted by nearly all paracrine cell types commonly detected in the TME 25, 26, and transfer diverse biomolecules including circRNAs, which participate in intercellular interactions when they are released and delivered into recipient cells 4. Some studies have illustrated the potential role of exosomal circRNAs in cancer progression, angiogenesis, chemoresistance and metabolism through regulating the TME 27-29.

## Functions of exosomal circRNAs in the TME

Certain innovative methods for investigating the biological foundations of exosome transport have been developed to monitor the dynamic communication of exosomes in different models 30-33. However, current isolation techniques of exosomes, including centrifugation, filtration, polymeric precipitation, and immunoaffinity separation 34, 35, often cause variations in downstream analysis results. Hence, there remains an urgent need to develop effective strategies for functional identification of exosomal cargoes (e.g., circRNAs) in different cancers.

**Figure 2:**
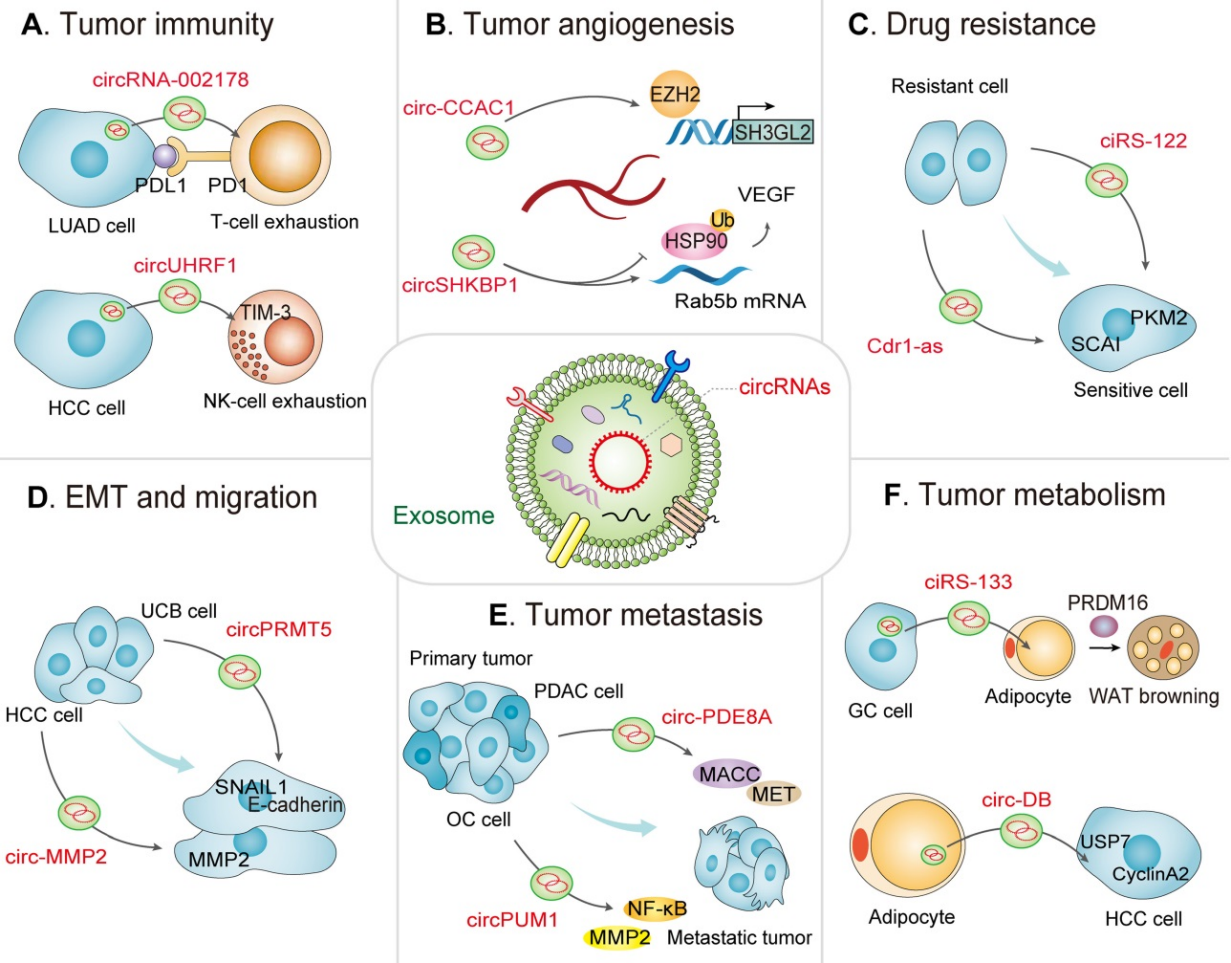
** Exosomal circRNA-mediated intercellular crosstalk in the TME.** A view of the molecular crosstalk pathways involving exosomal circRNAs in the TME, including (A) tumor immunity, (B) tumor angiogenesis, (C) drug resistance, (D) EMT and migration, (E) tumor metastasis, and (F) tumor metabolism.

In the TME, exosomes mediate the interplay between immune cells and tumor cells 36, 37. The TDEs play pivotal roles in the establishment of pre-metastatic niches, immune escape, tumor immune microenvironment, immune suppression, and immune surveillance 38. Notably, circRNAs can specifically interact with tumor-specific miRNAs or mRNAs in exosomes, functioning as new tumor antigens for mediating immune responses 39, 40. In the following sections, we focus on the expanding landscape of exosomal circRNAs in the TME, including tumor immunity, angiogenesis, drug resistance, EMT, tumor cell migration, tumor metastasis, as well as tumor metabolism (**Table 1 and Figure 2**).

### Exosomal circRNAs regulate tumor immunity

As the richest cellular components of the TME, immune cells have been reported as promising targets for their anti-cancer cytotoxic capabilities 41. Interactions among immune cells, cancer cells, and exosomes associated intercellular communication are important in tumor immunoregulation, which can create immunosuppressive environments fostering cancer development and progression 42, 43. Hence, identifying key regulators within this interplay may provide candidates for therapeutic interventions. Recently, dramatic dysregulation of many exosomal circRNAs in the TME were observed in diverse cancer types 8. Additionally, some exosomal circRNAs regulate immune cells including lymphocytes, macrophage, NK cells, and dendritic cells (DCs), thereby providing evidence for the functional roles of exosomal circRNAs in immune regulation.

As a major component of adaptive immunity, the majority of T lymphocytes (also called T cells) exhausted in the TME induce tumor immune escape 44. Programmed death ligand 1 (PD-L1, CD274), known as an essential immune checkpoint protein, is a crucial constituent of tumor immunosuppression that binds to programmed death 1 (PD-1) on T-cells 45. The ability of PD-L1/PD-1 to suppress T cell activation and to augment the immune tolerance of tumor cells is crucial to achieve tumor immune escape 46. Interestingly, multiple observations have exhibited that exosomal circRNAs severed as crucial regulators of immune escape. A recent study detected plasma exosomal circRNA-002178 in lung adenocarcinoma (LUAD) patients and suggested that circRNA-002178 facilitates PD-L1 expression by acting as miR-34 sponge in LUAD, thus inducing T-cell exhaustion. Therefore, circRNA-002178 could be attractive as a promising noninvasive biomarker for the early detection of LUAD, and could enhance PD-L1 expression to mediate interactions between cancer cells and T cells **(Figure 2A)** 47. Similarly, exosomal circ-0001068 is transferred into T cells, where it caused an upregulation of PD1 expression by sponging miR-28-5p in ovarian cancer (OC) 48. Furthermore, Hong *et al*. demonstrated that non-small-cell lung cancer (NSCLC) cells release PD-L1-containing exosomes that boost cell stemness and upregulate the resistance of NSCLC cells to cisplatin 49. NSCLC cells also induce the inactivation of CD8^+^ T cells in a delivered PD-L1-dependent manner. Furthermore, this finding indicates that circ-CPA4 positively mediates exosomal PD-L1, while NSCLC cells with circ-CPA4 deletion exhibit reactivation of CD8^+^ T cells in the co-culturing system. Therefore, deciphering exosomal circRNA-induced suppressive mechanisms of T cell activation will accelerate the discovery and development of novel immune checkpoint inhibitors for targeting complement-mediated immunoregulation.

Macrophages, the most prevalent immune cells in the TME, are an integral factor that hinders tumor progression and influences the antitumor immunity response 50. Exosomes can act as a communicator connecting tumor cells and macrophages 51. For instance, exosomal hsa-circ-0048117 has been reported to be upregulated in esophageal squamous cell carcinoma (ESCC). Exosomal hsa-circ-0048117 could be delivered to macrophages to induce M2 macrophage polarization, thereby remodeling the hypoxic microenvironment and regulating ESCC progression 52. In the TME, tumors are able to recruit and induce macrophages to develop into tumor-associated macrophages (TAMs) that facilitate tumor progression. For example, circFARSA is elevated in NSCLC cells and associated with the elevation of EMT and metastasis. Moreover, circFARSA derived from NSCLC cells induces tumor TAMs polarization to M2 phenotype by promoting PTEN ubiquitination and subsequent degradation, thereby activating the PI3K/AKT signaling pathway 53.

NK cells are the first line of defense for host immune surveillance and, as such exert pivotal effects on antitumor immunotherapy. Due to the fact that NK cells have no major histocompatibility complex limitations on the recognition and damage of target cells, accumulating immunostimulants can be created to enhance cell killing 54. Moreover, the density and activity of NK cells in the TME are correlated with prognoses of various cancers 55. NK cell-derived exosomal circRNAs also serve as crucial players in the innate antitumor immune response. For instance, Zhang *et al*. recently showed that exosomal circUHRF1 secreted by hepatocellular carcinoma (HCC) cells induce NK cell exhaustion, thus mediating resistance to anti-PD1 therapy. Additionally, high expression of circUHRF1 in plasma exosomes correlates with a reduced proportion of NK cells as well as NK cell tumor infiltration. Moreover, HCC-derived exosomal circUHRF1 is transferred into NK cells, sponging miR-449c-5p to upregulate TIM-3 expression in turn inducing NK cell exhaustion, thereby favoring immunosuppression and resistance to anti-PD1 immunotherapy in HCC, offering a therapeutic target for patients with HCC **(Figure 2A)** 27.

Dysregulation of tumor infiltrating lymphocytes (TILs) and neutrophils in the TME is associated with overall survival 56, 57. Pancreatic ductal adenocarcinoma (PDAC) is one of the most invasive and fatal forms of carcinomas with a low 5-year survival rate of 5%, due to of a high-risk of metastasis and recurrence 58, 59. Nevertheless, breakthroughs have been made in exosome-mediated circRNAs crosstalk in PDAC. For instance, Li *et al*. reported that PDAC cell-derived exosomal circ-PDE8A is related to lymphatic invasion and tumor progression, thereby activating the MACC/MET/ERK or AKT pathways by sponging miR-338 60. Intriguingly, another recent study showed that high expression of cancer-derived exosomal circPACRGL promotes colorectal cancer (CRC) proliferation and metastasis. Functional assays revealed that circPACRGL sponges miR-142-3p/miR-506-3p to promote transforming growth factor-β1 (TGF-β1) expression, thereby contributing to CRC cell proliferation, migration and invasion, and differentiation of N1 to N2 neutrophils 61. Currently, studies on the precise mechanisms of exosomal circRNAs in regulating immune cells are still in their infancy and more work is needed for further investigation to discover novel therapeutic targets.

### Exosomal circRNAs mediate tumor angiogenesis

Angiogenesis is a complex process by which tumors not only obtain adequate nutritional support but also remove metabolic waste and carbon dioxide 11. Overexpression of diverse angiogenic factors, as well as rapid growth of tumor cells in the TME, contribute to the development of vascular networks, thus leading to diverse structural and functional abnormalities 62, 63. Vascular endothelial growth factor (VEGF), a potent angiogenic factor, plays an essential role in both physiological and pathological angiogenesis 64. Meanwhile, cancer-associated endothelial cells are a significant component of the tumor stroma in the TME, that supports tumor neovasculature and blood vessel formation 70. Furthermore, certain inhibitors (such as bevacizumab) can markedly decrease the expression of angiogenic factors (VEGF), thereby inhibiting angiogenesis 65. Therefore, advancing our current understanding of the cellular and molecular mechanisms associated with tumor angiogenesis will enable the development of novel anti-angiogenic therapies 66.

Exosomal circRNAs can affect tumor angiogenesis by interacting with epigenetic regulators, thereby remodeling the TME to induce cancer initiation and progression. For instance, a recent study demonstrated that circ-CCAC1 expression is significantly upregulated in cancerous bile-resident EVs and tissues. Further analysis uncovered that cholangiocarcinoma (CCA)-derived EVs circ-CCAC1 is delivered to endothelial monolayer cells, thus destroying endothelial barrier integrity and promoting angiogenesis. Mechanistically, circ-CCAC1 upregulates cell leakiness via sequestering EZH2 in the cytoplasm, thereby increasing SH3GL2 expression to decrease intercellular junction proteins expression. Furthermore, *in vivo* studies revealed that elevated circ-CCAC1 levels in circulating EVs and cells facilitated both CCA tumorigenesis and metastasis (**Figure 2B**) 28.

Exosomal circRNAs also regulate angiogenesis by simultaneously acting as miRNA and RNA-binding protein (RBP) "sponges". For instance, current data provided by Xie *et al*. indicates that exosomes with elevated circSHKBP1 facilitate growth of cocultured gastric cancer (GC) cells. Mechanistically, circSHKBP1 acts as a competing endogenous RNA (ceRNA) for miR-582-3p to upregulate the expression of HUR, augmenting *VEGF* mRNA stability. Furthermore, circSHKBP1 directly interact with HSP90, thereby impeding the interaction between HSP90 and STUB1, subsequently suppressing ubiquitination of HSP90, and giving rise to the acceleration of GC development *in vivo* and *in vitro*. This study confirmed that exosomal circSHKBP1 modulated the miR-582-3p/HUR/VEGF axis to inhibit HSP90 degradation and facilitated the progression of GC (**Figure 2B**) 67. Likely, Lu* et al*. revealed that circ-RanGAP1 is significantly upregulated in plasma exosomes of preoperative GC patients and mediates the miR-877-3p/VEGFA axis, thereby promoting the migration and invasion of GC 68. Another study indicated that circFNDC3B-enriched exosomes can suppress CRC angiogenesis and liver metastasis via miR-97-5p/TIMP3 axis 69.

Exosome-mediated transfer of circRNAs can also modulate the permeability of endothelial cells, facilitating the dissemination and metastasis of cancer cells. Tumor metastasis is the major risk factor for cancer-associated death. Tumor endothelial cells obtain their specific characteristics in the TME, provoking the metastasis of tumor cells. Endothelial cells particularly serve as crucial regulators in the original stage of tumor metastasis 70. Recent studies have elucidated the molecular mechanism by which exosomal circRNAs derived from tumor cells are involved in the regulation of the permeability of endothelial cells in the TME. For example, the pancreatic cancer (PC) cell-derived exosomal circRNA IARS can enter the human microvascular vein endothelial cells (HUVECs) via plasma exosomes and destroy the endothelial tight junctions, thus elevating endothelial cell permeability and facilitating the establishment of a microenvironment suitable for tumor invasion and metastasis. Further analysis revealed that the molecular axis of circRNA IARS/miR-122/RhoA/F-actin was responsible for these functions. Therefore, the behavior of exosomal circRNAs provides new insights into prospective endothelial monolayer permeability and contributes to preventing early tumor cell metastasis via suppressing endothelial cell permeability, suggesting potential targets for anti-angiogenesis strategy 71.

In addition to VEGF, other angiogenic factors are also regulated by exosome-delivered circRNAs, which either directly or indirectly affect tumor angiogenesis. A recent study reported that the upregulated exosomal circRNA-100338 in metastatic HCC cells, which are transported from HCC cells to HUVECs, influenced the permeability, angiogenesis, and proliferation of recipient HUVECs. Mechanistically, internalized circRNA can directly interact with NOVA2, a RBP, thereby mediating vascular development and achieving cell-to-cell communication 72.

Currently, certain clinical trials with antiangiogenic strategies failed to show clinical benefit in a subset of cancers, the overall outcome was not as promising as initially hoped for improved anticancer therapy. Current studies have demonstrated that exosomal circRNAs manipulate angiogenesis via different mechanisms to establish a favorable microenvironment, thereby providing dynamic information about the pathologic state of the exosome-producing cells. Targeting exosomal circRNAs from different cell types of the TME could be used as alternatives to the existing antiangiogenic therapies.

### Exosomal circRNAs modulate drug resistance

Drug resistance is closely related to the TME. Exosomes, as key mediators of intercellular communication, are involved in drug resistance. Tumor and stromal cells in the TME can secrete drug-resistant exosomes containing distinct biomacromolecules including circRNAs. As a research hotspot in recent years, circRNAs secreted by exosomes exert multifunctional roles in the transfer of molecules related to drug resistance from primary cancer cells to recipient cells 6. Accumulating evidence has shown that the emerging function and mechanistic axis of circRNAs are related to drug resistance. Intrinsic and extrinsic factors can exert an influence on cancer cell resistance to chemoradiation 73. The extrinsic factors such as exosomal circRNAs in the TME can facilitate chemoradiation resistance and tumor recurrence, including the ECM, hypoxia, and the expression of angiogenic markers (VEGF and HIF1α) 74, 75. As a result, the roles of exosomal circRNAs in the drug resistance could lead to the identification of novel therapeutic targets to develop more effective therapeutic agents for cancer treatment. Li. *et al*. observed that circRNA *FLI1* exonic circular RNAs (FECRs) derived from serum exosomes was abnormally elevated in small cell lung cancer (SCLC) in comparison with control groups and was closely associated with the clinical response to chemotherapy and poor survival in SCLC patients. Mechanistically, *FLI1* exonic circular RNAs (FECRs) act as a new oncogenic driver via the miR-584-ROCK1 pathway, thus determining the metastatic phenotype in SCLC 76. This suggests a possible connection between exosomal circRNAs dysregulation and drug resistance. Moreover, exosomal circRNAs may function as intercellular signaling molecules to transfer drug-resistance properties from drug-resistant CRC cells to sensitive cells. A recent investigation identified ciRS-122 to be positively associated with chemoresistance in CRC. Exosomal ciRS-122 is delivered from chemoresistant cells to recipient cells. Further, ciRS-122 can function as a miR-122 sponge to elevate pyruvate kinase M2 (PKM2), thereby accelerating glycolysis and enhancing the resistance of sensitive CRC cells to oxaliplatin. Therefore, ciRS-122 might be a therapeutic target for the therapy of drug-resistant CRC (**Figure 2C**) 77. Regarding chemoresistance in CRC, Hon *et al*. reported that serum exosomal hsa_circ_0000338 derived from fluorouracil/oxaliplatin/leucovorin (FOLFOX)-resistant CRC cells was elevated in comparison with levels in sensitive cell-derived exosomes. Further investigation showed that hsa_ circ_0000338 knockdown elevated the viability of FOLFOX-resistant CRC cells following exposure to 5-fluorouracil (5-FU) 78. Similarly, another recent study on gliomas showed that exosomal circNFIX was highly expressed in the serum of temozolomide (TMZ)-resistant patients and was correlated with poor prognosis. Exosomal circNFIX is transferred from TMZ-resistant cells to targetable sensitive cells via the augmentation of cell migration and invasion as well as the suppression of cell apoptosis under TMZ exposure. Moreover, exosomal circNFIX promoted tumor growth, and its depletion elevated TMZ sensitivity in glioma cells by increasing miR-132 *in vivo* 29.

Moreover, exosomal circRNA-SORE is elevated in sorafenib-resistant HCC cells, while its depletion enhances the cell-killing ability of sorafenib. Mechanistically, circRNA-SORE interacts with YBX1, a master oncogenic protein that, inhibits the interplay between YBX1 and E3 ubiquitin ligase PRP19 thus impeding PRP19-induced YBX1 degradation. Furthermore, silencing of circRNA-SORE via siRNA can overcome sorafenib resistance in HCC mouse models 79. Zhao* et al*. revealed that Cdr1as is decreased in serum exosomes of cisplatin-resistant patients and mediates the miR-1270/SCAI axis, thereby increasing the sensitivity of OC cells to cisplatin (**Figure 2C**) 80. Research involving cisplatin resistance in NSCLC has recently revealed that hsa_circ_0014235 in exosomes derived from the serum is markedly upregulated in NSCLC and facilitates cisplatin chemoresistance, while modulating the miR-520a-5p/CDK4 pathway, thus increasing NSCLC development 81. Regarding drug resistance in chronic lymphocytic leukemia (CLL), Wu *et al*. found that CLL-derived exosomal mc-COX2 (mitochondrial genome-derived circRNAs [mc]) is highly expressed in plasma and is positively correlated with leukemogenesis and the deteriorating survival of CLL patients. The endogenous decrease of mc-COX2 can influence mitochondrial functions, suppressing cell proliferation and inducing cell apoptosis 82.

The aformentioned studies show that cancer-associated cells are reprogrammed in the TME by utilizing various exosomal circRNAs to induce resistance of tumor cells to anticancer drugs or radiotherapy. Hence, these identified exosomal circRNAs may also be potential therapeutic targets in overcoming drug resistance, enhancing the clinical benefits in patients.

### Exosomal circRNAs induce EMT and tumor cell migration

Accumulating evidence suggests that certain exosomal circRNAs play a diverse range of roles in EMT. Activation of EMT under pathological conditions plays crucial roles in tumor development and the initiation of metastasis and is involved in the procedure of transforming epithelial cells into mesenchymal cells, leading to tumor cell migration 83, 84. Here are some examples regarding exosomal circRNAs-induced aberrant EMT activation, tumor cell motility and invasiveness. Chen. *et al*. revealed that serum and urine exosomal circPRMT5 was aberrantly upregulated in patients with urothelial carcinoma of the bladder (UCB). Further studies revealed that circPRMT5 could sponge miR-30c to promote UCB cell EMT and enhance the expression of its target genes E-cadherin and SNAIL1, thus enabling the cells to be more invasive (**Figure 2D**) 85. Similarly, 97H-derived exosomal circ_MMP2 (hsa_circ_0039411) could be transmitted into L02 and HepG2 cells and its overexpression was correlated with a poor overall survival of HCC patients. Mechanistically, circ_MMP2 enrichment can abundantly sponge miR-136-5p to upregulate the expression of its host gene matrix metallopeptidase 2 (MMP2), thus effectively enhancing the EMT process and invasive abilities of HCC cells (**Figure 2D**) 86. Feng *et al*. reported that circIFT80 expression was highly elevated in serum exosomes derived from CRC tissues and cells compared with that in exosomes derived from the normal counterparts. Functional assays revealed that circIFT80 sponges miR-1236-3p to facilitate HOXB7 expression, thus promoting CRC cell growth, proliferation, migration, and invasion via aberrant activation of EMT transcription factors 87.

Accumulating evidence also suggests that message transmission is crucial for tumor formation, and it is likely that exosomal circRNAs function as intercellular modulators in the process of carcinogenesis. For example, exosomal circRNA-100284 was transferred from malignant transformed cells into surrounding normal cells, leading to the malignant transformation of the non-transformed cells induced by arsenite. Functional assays revealed that exosomal circRNA_100284 sponges miR-217 to increase the expression of enhancer of zeste homolog 2 (EZH2) and cyclin D1, thus accelerating the cell cycle progression and promoting the proliferation of recipient cells 88. A study reported that plasma exosomal circ-0000284 was upregulated in cholangiocarcinoma (CCA) patients compared to that in healthy controls. CCA cell-derived exosomal circ-0000284 was transported into surrounding cells, thus promoting the proliferation and migration of recipient cells and suppressing apoptosis via the miR-637/lymphocyte antigen 6 complex locus E (LY6E) axis 89. Moreover, Tian *et al*. confirmed that serum exosome-derived circRASSF2 levels were abnormally elevated in laryngeal squamous cell carcinoma (LSCC) patients. circRASSF2 sponges miR-302b-3p to affect IGF-1R expression, promoting progression of LSCC 90.

Since exosomal circRNAs can be delivered into recipient cells and induce functional responses and phenotypic changes in cancers, it is important to design and conduct more experiments to confirm the functional role and underlying mechanisms of action of exosomal circRNAs in the TME to discover novel noninvasive prospective biomarkers and therapeutic targets for cancers.

### Exosomal circRNAs mediate tumor metastasis

Tumor onset and metastasis are correlated with multiple oncogenes and involve multiple oncogenic signaling pathways 91. Exosomal circRNAs play important roles in cancer cell-to-cell crosstalk and the surrounding stroma, contributing to metastasis. Li *et al*. first reported that exosomal circ-PDE8A levels were highly elevated in PDAC tissue and were significantly correlated with higher tumor-node-metastasis (TNM) stages, lymphatic invasion, and a poor survival rate. Furthermore, circ-PDE8A facilitated tumor cell growth by increasing the expression of MET (a tyrosine kinase receptor), encoded by one of the vital oncogenes for a subclass of epithelial tumors, containing PDA. Importantly, tumor-derived exosomal circ-PDE8A could be delivered into blood circulation, thereby mediating MACC1 and tumor metastasis by sponging miR-338 by the MACC/MET/ERK or AKT axis (**Figure 2E**) 60. Guan *et al*. reported that circPUM1 could sponge miR-615-5p and miR-6753-5p and elevate the expression of NF-κB and MMP2, thus acting on peritoneal mesothelial cells and promoting OC metastasis (**Figure 2E**) 92. Similarly, another study confirmed that exosomal circNRIP1 could be transported between GC cells, thus promoting cell proliferation, migration, and invasion by sponging miR-149-5p to activate the AKT1/mTOR signaling pathway 93. These findings were also confirmed in breast cancer 94.

Exosomal circRNAs are known to exert critical effects on the development and metastasis of HCC. Wang *et al*. showed that the upregulation of serum exosomal circPTGR1 from patients with HCC was significantly linked to the clinical stage and prognosis. Further analysis revealed that exosomal circPTGR1 knockdown significantly inhibited tumor migration and invasion of non- and low-metastatic cell lines. Mechanistically, exosomal circPTGR1 can promote the development of HCC by sponging miR-449a 95. Another study indicated that the elevated level of circWHSC1 in OC facilitated tumorigenesis by serving as miR-145 and miR-1182 sponge and the presence of these miRNAs in exosomes promoted tumor metastasis by acting on the peritoneal mesothelium. Functional assays revealed that exosomal circWHSC1 can be transmitted to peritoneal mesothelial cells to facilitate peritoneal dissemination by upregulating the expression of mucin 1 (MUC1) in recipient cells, thus promoting OC progression 96. Taken together, the above-mentioned findings have corroborated the extensive and pivotal role of exosomal circRNAs in tumor metastasis by regulating TME. Therefore, exosomal circRNAs may act as potential therapeutic targets for management of tumor metastasis and provide new directions for cancer therapy. Further research on exosomal circRNAs is warranted to deepen our understanding of their detailed mechanisms of action *in vivo* as well as their potential clinical applications.

### Exosomal circRNAs affect tumor metabolism

Since reprogramming energy metabolism is regarded as a new hallmark of cancer, tumor metabolism has attracted increasing attention in cancer research. To date, several metabolic interventions, including those associated with fatty acid oxidation and the respiratory complex I inhibitor, metformin 97, 98, have been successfully combined with immunotherapeutic agents to modulate anticancer effects 99. Growing knowledge regarding the metabolism of tumor cells in the TME has facilitated the design of novel therapies that are safe and effective for cancer patients 100. In fact, multiple therapeutic agents have been shown to affect tumor cell metabolism, glutaminolysis aerobic glycolysis, the adipose tissue microenvironment, as well as other metabolic targets 101-104. New therapeutic strategies are also being developed to target metabolism of stromal and immune cells 105, 106.

As a key example of the metabolic interplay, adipocytes can offer nutrients such as alanine and lipids to the TME, which support malignant cells 107, 108. For instance, Zhang *et al*. reported that the adipocyte-derived exocirc-deubiquitination (exo-circ-DB) can sponge miR-34a to activate the USP7/CyclinA2 signaling pathway, thus boosting HCC tumorigenesis and metastasis while reducing DNA damage (**Figure 2F**) 109. Zhang *et al*. revealed that plasma-derived exosomal ciRS-133 (circ-0010522) levels are elevated in GC patients and are closely related to the browning of white adipose tissue and cancer-related cachexia. Further studies reported that exosomal ciRS-133 is delivered into preadipocytes, where it inhibits miR-133 and activates PR domain containing protein 16 (PRDM16), thus facilitating white adipose browning, aggravating tumor cachexia, and increasing oxygen consumption in GC patients, thereby highlighting the crucial role of exosomal circRNAs in the pathologic process (**Figure 2F**) 110.

Aerobic glycolysis is a hallmark of tumor cells, which allows them to meet their high demand for energy required for tumor growth and maintenance 111. Meanwhile, exosomal circ-MEMO1 promotes the progression and glycolytic metabolism of NSCLC by modulating the miR-101-3p/KRAS axis 112. Exosomal circRNAs also regulate tumor metabolism by inducing cell death, such as autophagy. Zhang *et al*. recently identified that exosomal circNRIP1 can be transferred between GC cells and promote EMT and tumor metastasis *in vivo*. Moreover, the exosomal circNRIP1/AKT1/mTOR axis can promote GC metastasis by altering metabolism and autophagy 93.

Tumor cells and stromal cells in the TME generally restrict the entry of nutrients and oxygen, inducing a hypoxic environment 113. Accumulating evidence suggests that the hypoxic TME is closely involved in multiple "hallmarks of cancer" 114, 115. Accordingly, studies are underway to assess hypoxia-regulated cancer biology, including exosome-mediated crosstalk in the TME. The TDEs, which are abundant in the TME, transport tumoral signaling to both stromal cells and tumor cells and exert an essential influence on numerous pathological scenarios 116. As transcriptional regulators, HIFs acts as a pivotal regulator in mediating the proliferation and metastasis of tumor cells and the responses of the TME via activating the transcription of downstream oncogenes, including hypoxia-responsive elements, and mediating diverse signaling pathways 117, 118. The exosomal circRNA-mediated cell interplay within hypoxic TME has been widely studied. Recently, Yang *et al*. reported that hypoxic cell-derived exosomal circ-133 is transferred into normoxic cells and regulates the E-cadherin membrane distribution through activation of the miR-133a/GEF-H1/RhoA signaling pathway, thus promoting CRC metastasis 119.

Carcinoma-associated fibroblasts (CAFs) plays a pivotal role in the TME at depositing and remodeling ECM 120. Hypoxia is one of the key components in the TME that greatly affects fibroblast activation. For instance, a recent study demonstrated that circHIF1A (circ_0032138) expression is significantly elevated in the exosomes from hypoxic CAFs. Further analysis uncovered that exosomal circHIF1A from hypoxic CAFs could promote breast cancer stemness through miR-580-5p/CD44 axis 121. Another study found that exosomal circSLC7A6 from CAFs could promote CRC progression. Additionally, matrine suppressed CRC tumorigenesis by modulating CXCR5 and blocking the secretion of exosomal circSLC7A6 122. These findings indicated that the specific role of CAFs in tumor progression could be a promising therapeutic strategy for the development of anti-tumor therapy.

Together, exosomal circRNAs execute a broad repertoire of functions to influence hypoxia and adipocyte-related regulators in the TME. As the TME contains different cell types with flexible metabolic programming, well-designed experiment will be required to unveil the metabolic demands, adaptations, and interactions among various cancer cells. Given that metabolic changes related to the TME contribute to cancer development and progression, targeting metabolic dysfunction through the use of different strategies may reshape the TME to improve efficacy of oncology drugs.

## Potential clinical applications of exosomal circRNAs in cancer therapy

**Figure 3:**
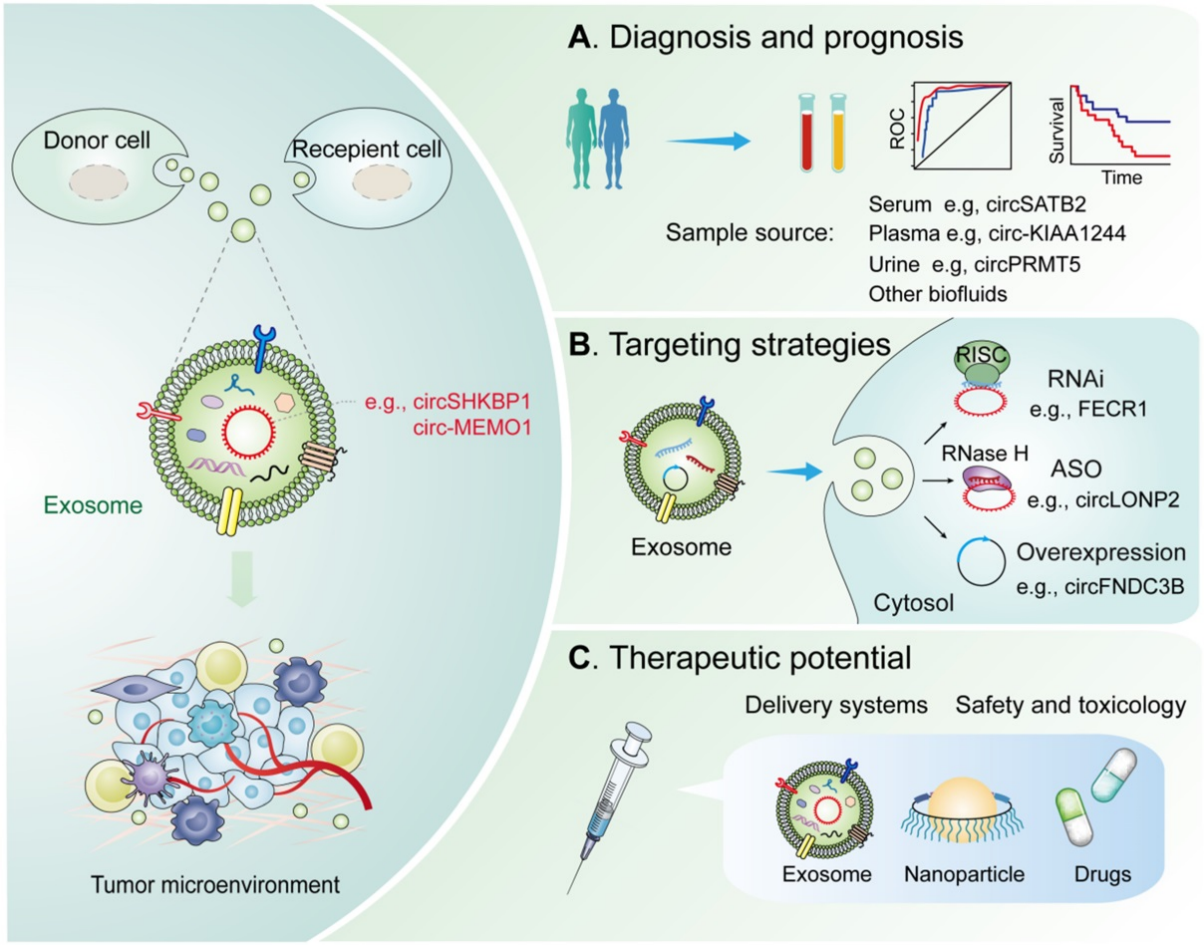
** Potential clinical applications of exosomal circRNAs in the TME.** Diagnostic and therapeutic potential of exosomal circRNAs in the TME. (A) Diagnosis and prognosis. These exosomal circRNAs can be measured from liquid biopsy samples and are novel noninvasive diagnostic and prognostic biomarkers. (B) Targeting strategies. Knockdown or overexpression technologies can be used to target circRNAs in the cytoplasm and nucleus. (C) Therapeutics. Exosomal circRNAs can be delivered via nanoparticles or antibody/receptor conjugates *in vivo*. Abbreviation: RNAi, RNA interference; ASO, antisense oligonucleotides; RISC, RNA-induced silencing complex; RNase H, Ribonuclease H.

Characterizing the underlying mechanism associated with exosomal circRNAs in suitable models could provide further evidence for their potential clinical application in cancer therapy. Current research has established that exosomal circRNAs exhibit great potential as potential biomarkers and therapeutic targets in cancer treatment. The application of biomarkers in cancer therapy will allow oncologists to better optimize therapeutic strategies for cancer patients and practice precision medicine in clinical settings. In the following sections, we discuss how exosomal circRNAs could be utilized as potential biomarkers for diagnosis, prognosis, and treatment monitoring for different cancer types (**Figure 3**).

### Diagnostic potential of exosomal circRNAs

Accumulating evidence indicates that multiple circRNAs involved in the regulation of the TME are dysregulated in diverse cancers 123, 124. Exosomal circRNAs are critical regulators of healthy and diseased states and may represent valuable biomarkers for diagnoses and prognoses of multiple cancers (**Figure 3A**).

The use of exosomal circRNAs as potential biomarkers in lung cancer has been proposed. For instance, Li *et al*. reported that serum Friend leukemia virus integration 1 (FLI1) is aberrantly upregulated in SCLC and is inextricably linked to poor survival and the clinical response to chemotherapy 76. Similarly, Xian *et al*. reported that circ_0007761, circ_0056285, and circ_0047921 can be used for diagnosis of NSCLC. In differentiating between NSCLC cases and healthy controls, a panel of the above-mentioned circRNAs possessed an AUC of 0.919 (95% CI, 0.877-0.962) in the validation set and an AUC of 0.926 (95% CI, 0.895-0.956) in the training set. Moreover, circ_0047921 can effectively discriminate between NSCLC patients from chronic obstructive pulmonary disease (COPD) controls (AUC, 0.890; 95% CI, 0.831-0.948), whereas the combination of circ_0056285 and circ_0007761 could differentiate between NSCLC patients and tuberculosis controls (AUC, 0.820; 95% CI, 0.739-0.902). Similar findings were found for these circRNAs in distinguishing early-stage NSCLC patients from COPD controls, tuberculosis controls, or healthy controls for early diagnosis of NSCLC. Further, circ_0056285 expression was linked to lymph node metastasis and clinical stage 125. Zhang *et al*. showed that serum exosomal circSATB2 was highly elevated in lung cancer patients with high specificity and sensitivity for clinical detection. Interestingly, exosomal, circSATB2 is correlated well with lung cancer metastasis, suggesting it as a prognostic biomarker for NSCLC 126.

Exosomal circRNAs as valuable biomarkers for the diagnosis and prognosis of GC have been reported. For example, Shao *et al*. reported that the plasma exosome-derived hsa_circ_0065149 expression levels are reduced in early-stage GC patients. Moreover, exosomal hsa_circ_0065149 expression has higher sensitivity and specificity in early-stage GC screening compared to the traditional clinical biomarkers 127. More importantly, exosomal circ-KIAA1244 may be stable in plasma due to the protection of exosomes from degradation. Hence, exosomal circ-KIAA1244 derived from GC might be an exploitable circulating biomarker for GC screening 128.

CircRNAs associated with TDEs might represent promising biomarkers in CRC as well. For instance, the expression of serum exosomal hsa_circ_0004771 is markedly elevated in CRC patients, compared with that in healthy individuals and benign intestinal disease (BID) patients, and is closely associated with cancer metastasis and TNM stage in CRC patients. Moreover, the area under the ROC curves (AUCs) of circulating exosomal hsa-circ-0004771 can effectively differentiate benign intestinal diseases (BIDs), stage I/II CRC patients, and CRC patients from healthy controls. Notably, the AUC value that differentiate stage I/II CRC patients from patients with BIDs was 0.816 (95% CI, 0.728-0.9) 129. Additionally, serum exosomal circFMN2 levels have been shown to be significantly upregulated in CRC patients, contributing to the progression of CRC via the miR-1182/hTERT signaling pathway 130. Other studies have indicated that numerous circRNAs are markedly downregulated in *KRAS* mutant cells and can be transmitted by exosomes secreted by tumor cells 131. In CRC cell lines, many circRNAs were also detected in secreted EVs including exosomes, indicating that EV or exosomal circRNAs may act as potential cancer biomarkers 131.

Recently, studies have reported that exosomal circRNAs are also potential diagnostic and prognostic biomarkers for HCC patients. For example, plasma exosomal hsa_circ_0051443 is expressed at lower levels in HCC patients compared to healthy controls. Normal cell-derived exosomal hsa_circ_0051443 is transmitted into HCC cells and suppresses malignant characteristics, thereby modulating intercellular crosstalk during HCC carcinogenesis 132. Analogously, in another report, Zhong *et al*. identified eight extracellular vesicle long RNAs (exLRs), including mRNA, circRNA, and lncRNA, that are potential biomarkers with high diagnostic performance in the testing cohort (AUC, 0.9627; 95% CI, 0.9263-0.9991), the training cohort [AUC, 0.9527; 95% CI, 0.9170-0.9883], and the validation cohort (AUC, 0.9825; 95% CI, 0.9606-1).Thus, numerous exLRs in human plasma are capable of serving as potential biomarkers for diagnosis of cancers 133.

Currently, certain exosomal circRNAs have been confirmed as biomarkers in papillary thyroid carcinoma (PTC). For instance, Wu *et al*. reported that serum exosomal levels of circRASSF2 and circ_0006156 (circFNDC3B) are elevated in PTC patients and mediate PTC progression via the miR-1178/TLR4 pathway, thereby highlighting exosomal circRNAs as biomarkers for diagnosis, prognosis and treatment of PTC 134, 135.

To date, multiple diagnostic clinical trials are underway to validate the utility of emerging exosome-based biomarkers for diagnosis and prognosis as well as treatment prediction in various types of cancer. For example, RNA profiling from circulating exosomes can be used as a biomarker for lung metastases of primary high-grade osteosarcoma (NCT03108677). For updated and complete information on exosomal circRNA clinical trials, please refer to ClinicaTrials.gov database.

Together, exosomal circRNAs as valuable biomarkers for diagnosis, prognosis and treatment of diverse cancers provide insights into the normal or pathological state of circRNA-producing cells. Due to their high stability, it is important to validate exosomal circRNAs in serum/plasma or other biofluids as biomarkers for diagnosis, prognosis, and treatment prediction of cancers. However, the main limitation of exosomal circRNAs as cancer biomarkers is that the utility of these potential biomarkers has not yet been validated with large sample size in multi-centre trails. Given the heterogeneity of tumor-associated circRNAs in different human populations, it is difficult to compare results between study groups from populations in different geographical locations. Therefore, well designed clinical trials should be conducted to validate diagnostic and prognostic value of these exosomal circRNAs in a wide range of cancers.

### Strategies to target exosomal circRNAs

Considering the crucial biological functions of exosomal circRNAs in tumor progression and metastasis, as the exosomal circRNAs may prove to be promising diagnostic and therapeutic options in cancer therapy. Hence, many ongoing studies have been designed to either regulate exosome production or block exosome uptake pathways. An increasing amount of research has emphasized that circRNAs may represent oncogenic drivers or tumor-suppressive mediators in a variety of cancers 136, 137. Currently, studies have focused on the clinical utility of circRNAs as therapeutic targets of cancer 136, 138. Effective techniques for gene overexpression or knockdown may provide clues for the therapeutic targeting of circRNAs.

RNA-based therapeutic strategies primarily include RNA interference (RNAi) and antisense oligonucleotides (ASOs), which can be designed to target a large and heterogeneous class of transcripts (Figure 3B) 139. For oncogenic circRNAs, specific shRNAs or siRNAs targeting the back-splice junction, which can be interfered with by homologous linear mRNA expression, were utilized to reach circRNA-specific knockdown 140. In terms of intron circularized circRNAs, researchers have carefully designed complementary paired siRNAs to target intron region sequences to destroy RNA formation, inducing circRNA knockdown 141. Serum exosomal FECR1 is a novel oncogenic circRNA in small cell lung cancer, which targeting it with shRNA can inhibit tumor metastasis in nude mice and improve overall survival 76. ASOs have shown promise in targeting of ncRNAs in patients with cancers. For example, circLONP2 is increased in metastasis-initiating CRC subgroups, acting as a key metastasis driver of CRC. Meanwhile, targeting circLONP2 by ASOs restricts metastasis to foreign organs, thereby functioning as a promising anti-metastatic therapeutic approach 142. For tumor-suppressive circRNAs, overexpression vectors fostering back-splicing consist of flanking introns with reverse complementary sequences and circRNA-forming exons 143. Recent preclinical studies have highlighted protein-coding genes derived functional circRNAs might provide a new repertoire of candidates for RNA-based therapeutics such as circRNAs. LoVo cell-derived exosomes with circFNDC3B overexpression have been used to treat CRC in animal model 69. Engineered exosome-based therapy has been applied in a phase I clinical trial for the treatment of pancreatic cancer patients with KrasG12D mutation (NCT03608631). Together, more well-designed circRNA-specific therapies are expected in the near future.

Engineered exosomes are designed for clinical applications by artificially optimizing a combination of specific cargo, including RNA-based therapy and tumor drugs. As they deliver specific bioactive molecules identified in the membrane of their progenitor cells, exosomes can be improved through the application of specific factors capable of targeting their transport to the TME. For instance, immature DC-derived exosomes modified with tumor targeting ligands such as the αv integrin-specific iRGD peptide can be employed for the transportation of DOX to tumors. Therefore, this method represents a promising strategy for targeted tumor therapy 144. A previous report has confirmed that the transmission of miRNA or siRNA payloads through exosomes is a prospective clinical tool in exosome-mediated therapies for various cancer treatments 5. Together, exosomal circRNAs (e.g., circ-MEMO1) might be a promising therapeutic target, while targeting circRNAs might be a novel therapeutic approach for cancer therapy. Development of more advanced technology that allows for the clinical applications of RNA-based therapy for cancer treatment remains warranted.

### Therapeutic potential of exosomal circRNAs

Current studies have identified exosomes as delivery tools for circRNA-targeting agents and circRNA expression vectors (**Figure 3C**) 145, 146. Using exosomes, one study has delivered a siRNA targeting ciRS-122, ultimately inhibiting CRC glycolysis and reverse resistance to oxaliplatin in mice 77. Another example used exosomes as delivery tools for circRNA expression vector showed that delivery of engineered rabies virus glycoprotein-circSCMH1-EVs promoted functional recovery in both and nonhuman primate models 147. Therefore, exosome engineering is a prospective tool in circRNA-based therapeutic strategy with clinical application; however, certain limitations such as the limited efficacy of the exosome route of injection or the quantity of exosomes administered, cell line employed, or the site of infusion remain to be addressed 148. Therefore, new strategies are necessary to advance the clinical potential of exosome-based therapeutics.

Exosomes are more biocompatible than man-made nanoparticles for drug and RNA delivery, which have been well demonstrated in cancer therapy 149. Although exosomes may be more biocompatible than synthetic nanoparticles, this system faces multiple challenges as a delivery system due to difficulty in large-scale production and loading of RNA payloads. Nevertheless, human red blood cells (RBC) are being considered an ideal source due to safety and abundance. Additionally, RBC-derived EVs or exosomes lack both nuclear and mitochondrial DNA, while on the other hand, RBCs have been used safely and routinely for blood transfusions. Therefore, RBC-derived EVs or exosomes could be used as a natural delivery system of therapeutic RNAs for cancer therapy 150. Hence, RBC derived EVs or exosomes act as natural carriers of anticancer agents (e.g. circRNAs) and may represent an ideal delivery system because of their negligible antigenicity, minimal cytotoxicity. The development of standardized protocols and proper analytical approaches are crucial to improve the reproducibility in exosome research, thereby ensuring the applicable translation of exosomes in anticancer therapeutics 151. Development of specific and effective strategies to target circRNAs *in vivo* will accelerate research progress of circRNA-based therapeutics.

## Concluding remarks and future perspectives

It is a hot research areas to investigate the roles of circRNAs in the TME, including in immune escape, angiogenesis, drug resistance, EMT, tumor metastasis, and tumor metabolism. Accumulating evidence suggested that the roles of exosomal circRNAs in the TME are involved in diverse molecular mechanisms such as serving as miRNA sponges, binding to proteins or DNAs, etc. Potential clinical applications of exosomal circRNAs in cancer diagnosis and prognosis are also being explored.

Recently, advances in the functions and mechanisms of exosomal circRNAs hold great promises for diagnostic and therapeutic applications for cancers. However, there are a series of key questions that remain to be answered: (i) Could exosomal circRNAs in body fluids be a novel source of candidate biomarkers for cancer diagnosis? Currently, it is too early to determine that since the research on exosomal circRNAs is still in its infancy and much more research is warranted to explore their potential application in cancer diagnosis. Nevertheless, circRNAs have been demonstrated to have better stability, promoting their storage, isolation and detection. (ii) If targeting circRNAs could be used as a novel therapeutic strategy in cancer therapy? Given that the current circRNAs studies have only been conducted in two-dimensional cell culture models, which lack the TME, the available data should be further verified using three-dimensional tumor organoid models. (iii) How to develop appropriate preclinical models to assess the safety, pharmacokinetic, and pharmacodynamic characteristics for clinical dose predictions? Various *in vitro* experimental strategies should be adopted to achieve this including the stability test of exosomal circRNAs under light and different temperature, the cytotoxicity test of exosomal circRNAs, and pharmacokinetic investigation using *in vitro* and *in vivo* models 152.

Exosomes have emerged as new organelles of interest in cancer diagnostics and treatment 153. Although some research progress has been made in the roles of exosomal circRNAs in the TME, there is a gap between basic findings and clinic application for diagnostic and prognostic biomarkers, and therapeutic target as well. Further large-scale, multicentered prospective cohort clinical studies are warranted to confirm the potential utility of exosomal circRNA in development of new therapy to improve cancer patient outcomes.

## Figures and Tables

**Table 1:** The emerging roles of exosomal circRNAs in the TME.

Exosomal circRNA	Source cell	Cancer types	Expression	Molecular axis	Functions	Ref.
**Exosomal circRNAs and tumor immunity**						
circRNA-002178	Lung adenocarcinoma cells	Lung adenocarcinoma	Up	circRNA-002178/miR-34/PDL1/PD1	Immune escape, T-cell exhaustion	47
circ-0001068	Ovarian cancer	Ovarian cancer	Up	circ-0001068/miR-28-5p /PD1	Immune escape, induce PD1 expression	48
circ-CPA4	Non-small cell lung cancer cells	Non-small cell lung cancer	Up	circ-CPA4/let-7 miRNA/PD-L1	Immune escape	49
circUHRF1	Hepatocellular carcinoma cells	Hepatocellular carcinoma	Up	circUHRF1/miR-449c-5p/TIM-3/anti-PD1	NK cell exhaustion	27
circ-PDE8A (hsa_circ_0036627)	Pancreatic ductal adenocarcinoma	Pancreatic ductal adenocarcinoma	Up	circ-PDE8A/miR-338/MACC1/MET	Lymphatic invasion and tumor progression, tumor infiltrating lymphocytes	60
circPACRGL	Colorectal cancer	Colorectal cancer	Up	circPACRGL/miR-142-3p/miR-506-3p-TGF-β1	Promote proliferation, migration and invasion, differentiation of N1 to N2 neutrophils.	61
hsa-circ-0048117	Hypoxia pre-challenged esophageal squamous cell carcinoma cells	Esophageal squamous cell carcinoma	Up	hsa-circ-0048117/miR-140	Promote M2 macrophage polarization	52
circFARSA	Non-small cell lung cancer	Non-small cell lung cancer	Up	circFARSA/PTEN/PI3K/AKT/eIF4A3	Promote M2 macrophage polarization	53
**Exosomal circRNAs and tumor angiogenesis**						
circ-CCAC1	Cholangiocarcinoma	Cholangiocarcinoma	Up	circ-CCAC1/miR-514a-5p/ YY1	Tumor angiogenesis by interacting with epigenetic regulators	28
circSHKBP1(hsa_circ_0000936)	Gastric cancer	Gastric cancer	Up	circSHKBP1/miR-582-3p/HUR/VEGF	Angiogenesis by acting as RBPs and miRNA "sponges"	67
circ-RanGAP1	Gastric cancer	Gastric cancer	Up	circ-RanGAP1/miR-877-3p/VEGFA	Regulate the expression of VEGFA by acting as specific miRNA “sponges”	68
circFNDC3B	Colorectal cancer	Colorectal cancer	Down	circFNDC3B/miR-97-5p/TIMP3	Inhibit angiogenesis and progression.	69
circ-IARS	Pancreatic cancer	Pancreatic cancer	Up	circRNA IARS /miR-122/RhoA /F-actin	Elevate endothelial cell permeability, facilitate tumor metastasis and invasion.	71
circRNA-100,338	Hepatocellular carcinoma cells	Hepatocellular carcinoma	Up	circRNA-100,338/ NOVA2	Influence the permeability, angiogenesis and proliferation	72
**Exosomal circRNAs and therapeutic resistance**						
ciRS-122	Oxaliplatin-resistant colorectal cancer cells	Colorectal cancer	Up	ciRS-122/miR-122/PKM2	Promote glycolysis to induce chemoresistance	77
circ_0000338	FOLFOX-resistant colorectal cancer cells	Colorectal cancer	Up	——	Induce chemoresistance	78
circNFIX	Temozolomide-Resistant glioma cells	Glioma	Up	circNFIX/miR-132.	Enhance temozolomide resistance, diagnostic biomarker (AUC 0.885) and prognostic biomarker	29
circRNA-SORE	Sorafenib-resistant hepatocellular carcinoma cells	Hepatocellular carcinoma	Up	circRNA-SORE/YBX1/PRP19	Spread sorafenib resistance	79
Cdr1as	Cisplatin-resistant ovarian cancer	Ovarian cancer	Down	Cdr1as/miR-1270/SCAI	Suppress cisplatin resistance	80.
hsa_circ_0014235	Non-small cell lung cancer	Non-small cell lung cancer	Up	circ_0014235/miR-520a-5p/CDK4	Promote cisplatin resistance	81
mc-COX2	Chronic lymphocytic leukemia	Chronic lymphocytic leukemia	Up	——	Strengthen drug resistance	82
**Exosomal circRNAs and migration and epithelial mesenchymal transition (EMT)**						
circPRMT5	Urothelial carcinoma of the bladder	Urothelial carcinoma of the bladder	Up	circPRMT5/miR-30c/SNAIL1/E-cadherin	Epithelial mesenchymal transition (EMT), biomarker of metastasis	85
circ_MMP2 (hsa_circ_0039411)	Hepatocellular carcinoma cells	Hepatocellular carcinoma	Up	circ_MMP2/miR-136-5p/MMP2	Enhance the EMT process and invasion	86
circIFT80	colorectal cancer	Colorectal cancer	Up	circIFT80/miR-1236-3p/HOXB7	Promote growth, proliferation, migration, and invasion via EMT	87
circRNA_100284	Arsenite-transformed human hepatic epithelial cells	Carcinogenesis induced by Arsenic	Up	circRNA_100284/microRNA-217	Accelerate the cell cycle and promote cell proliferation	88
circ-0000284	Cholangiocarcinoma cells	Cholangiocarcinoma	Up	circ-0000284/miR-637/LY6E	Promote proliferation and migration, suppress apoptosis	89
circRASSF2	Laryngeal squamous cell carcinoma	Laryngeal squamous cell carcinoma	Up	circRASSF2 /miR-302b-3p/IGF-1R	Enhance proliferation, migration, and invasion	90
**Exosomal circRNAs and tumor metastasis**						
circ-PDE8A (hsa_circ_0036627)	Pancreatic ductal adenocarcinoma	Pancreatic ductal adenocarcinoma	Up	circ-PDE8A/miR-338/MACC1/MET	Lymphatic invasion and tumor progression, tumor infiltrating lymphocytes	60.
circPUM1	Ovarian cancer	Ovarian cancer	Up	circPUM1/miR-615-5p/ miR-6753-5p/NF-κB/ MMP2	Act on peritoneal mesothelial cells and promote metastasis	92
circNRIP1	Gastric cancer	Gastric cancer	Up	circNRIP1/miR-149-5p/AKT1/mTOR	Promote EMT and metastasis	93
circPTGR1	Hepatocellular carcinoma	Hepatocellular carcinoma	Up	circPTGR1/miR449a/MET	Promote metastasis and invasion	95
circWHSC1	Ovarian cancer	Ovarian cancer	Up	circWHSC1/miR-145 /miR-1182/MUC1 /hTERT	Induce tumor metastasis through acting on peritoneal mesothelium	96
**Exosomal circRNAs and tumor metabolism**						
circ-DB	Adipocytes	Hepatocellular carcinoma	Up	circ-DB/miR-34a/USP7/Cyclin A2	Promote tumorigenesis and metastasis	109
ciRS-133 (circ-0010522)	Gastric cancer cells	Gastric cancer	Up	ciRS-133/miR-133/ PRDM16	Promote white adipose browning, aggravate tumor cachexia and increase oxygen consumption	110
circ-MEMO1	Non-small cell lung cancer	Non-small cell lung cancer	Up	circ-MEMO1/miR-101-3p/KRAS	Facilitate progression and glycolysis	112
circ-133	Hypoxic colorectal cancer cells	Colorectal cancer	Up	circ-133 /miR-133a/GEF-H1/RhoA	Promote tumor metastasis and migration	119
circHIF1A	Hypoxic cancer-associated fibroblasts	Breast cancer	Up	circHIF1A/miR-580-5p/CD44	Promote breast cancer cell proliferation and stemness	121
circSLC7A6	Cancer-associated fibroblasts	Colorectal cancer	Up	circSLC7A6/ CXCR5	Promote colorectal cancer cell proliferation and invasion	122

## Abbreviations

ASOs: antisense oligonucleotides
AUCs: area under the ROC curves
CCA: cholangiocarcinoma
ceRNA: competing endogenous rna
CircRNA: circular RNA
ciRNAs: intronic circRNAs
CLL: chronic lymphocytic leukemia
CRC: colorectal cancer
DCs: dendritic cells
GC: gastric cancer
ecircRNAs: exonic circRNAs
EIciRNAs: exonic-intronic circRNAs
EVs: extracellular vesicles
exLRs: extracellular vesicle long RNAs
EZH2: enhancer of zeste homolog 2
HCC: hepatocellular carcinoma
HUVECs: human microvascular vein endothelial cells
LSCC: laryngeal squamous cell carcinoma
LUAD: lung adenocarcinoma
LY6E: lymphocyte antigen 6 complex locus E
MMP2: matrix metallopeptidase 2
MUC1: mucin 1
MVB: multivesicular body
NcRNAs: noncoding RNAs
NK: natural killer
NSCLC: non-small-cell lung cancer
PC: pancreatic cancer
PD-L1: programmed death ligand 1
PDAC: pancreatic ductal adenocarcinoma
OC: ovarian cancer
PKM2: pyruvate kinase M2
PRDM16: pr domain containing protein 16
PTC: papillary thyroid carcinoma
RBP: RNA-binding protein
RIG-I: retinoic acid-inducible gene I
SCLC: small cell lung cancer
SRY: sex-determining region Y
TDEs: tumor-derived exosomes
TILs: tumor infiltrating lymphocytes
TGF-β1: transforming growth factor-β1
TME: tumor microenvironment
TMZ: temozolomide
TNM: tumor-node-metastasis
tricRNAs: circRNAs produced from tRNAs
Tregs: regulatory t cells
UCB: urothelial carcinoma of the bladder
VEGF: vascular Endothelial growth factor
